# Single-Chain Variable Fragment-Based Bispecific Antibodies: Hitting Two Targets with One Sophisticated Arrow

**DOI:** 10.1016/j.omto.2019.02.004

**Published:** 2019-03-23

**Authors:** Raoufeh Ahamadi-Fesharaki, Abolfazl Fateh, Farzam Vaziri, Ghasem Solgi, Seyed Davar Siadat, Fereidoun Mahboudi, Fatemeh Rahimi-Jamnani

**Affiliations:** 1 Department of Immunology, School of Medicine, Hamadan University of Medical Sciences, Hamadan, Iran; 2 Human Antibody Lab, Innovation Center, Pasteur Institute of Iran, Tehran, Iran; 3 Department of Mycobacteriology and Pulmonary Research, Microbiology Research Center, Pasteur Institute of Iran, Tehran, Iran; 4 Biotechnology Research Center, Pasteur Institute of Iran, Tehran, Iran

**Keywords:** monoclonal antibody, single-chain variable fragment, bispecific antibody, cancer, HIV-1, bacteria, ESKAPE, autoimmune diseases

## Abstract

Despite the success of monoclonal antibodies (mAbs) to treat some disorders, the monospecific molecular entity of mAbs as well as the presence of multiple factors and pathways involved in the pathogenesis of disorders, such as various malignancies, infectious diseases, and autoimmune disorders, and resistance to therapy have restricted the therapeutic efficacy of mAbs in clinical use. Bispecific antibodies (bsAbs), by concurrently recognizing two targets, can partly circumvent these problems. Serial killing of tumor cells by bsAb-redirected T cells, simultaneous blocking of two antigens involved in the HIV-1 infection, and concurrent targeting of the activating and inhibitory receptors on B cells to modulate autoimmunity are part of the capabilities of bsAbs. After designing and developing a large number of bsAbs for years, catumaxomab, a full-length bsAb targeting EpCAM and CD3, was approved in 2009 to treat EpCAM-positive carcinomas besides blinatumomab, a bispecific T cell engager antibody targeting CD19 and CD3, which was approved in 2014 to treat relapsed or refractory acute lymphoblastic leukemia. Furthermore, approximately 60 bsAbs are under investigation in clinical trials. The current review aims at portraying different formats of the single-chain variable fragment (scFv)-based bsAbs and shedding light on the scFv-based bsAbs in preclinical development, different phases of clinical trials, and the market.

## Main Text

High-affinity and specific binding to an antigen and effector functions, both resulting from a particular structure, make the antibody as one of the most compelling components of the immune system.^1^, ^2^, ^3^ Besides, good drug-like properties, such as solubility, stability, and prolonged half-life, led to the success of the therapeutic monoclonal antibodies (mAbs) in the clinics; therefore, a large number of antibodies or related products are either approved or under investigation in clinical trials.^1^, ^4^

**Figure 1:**
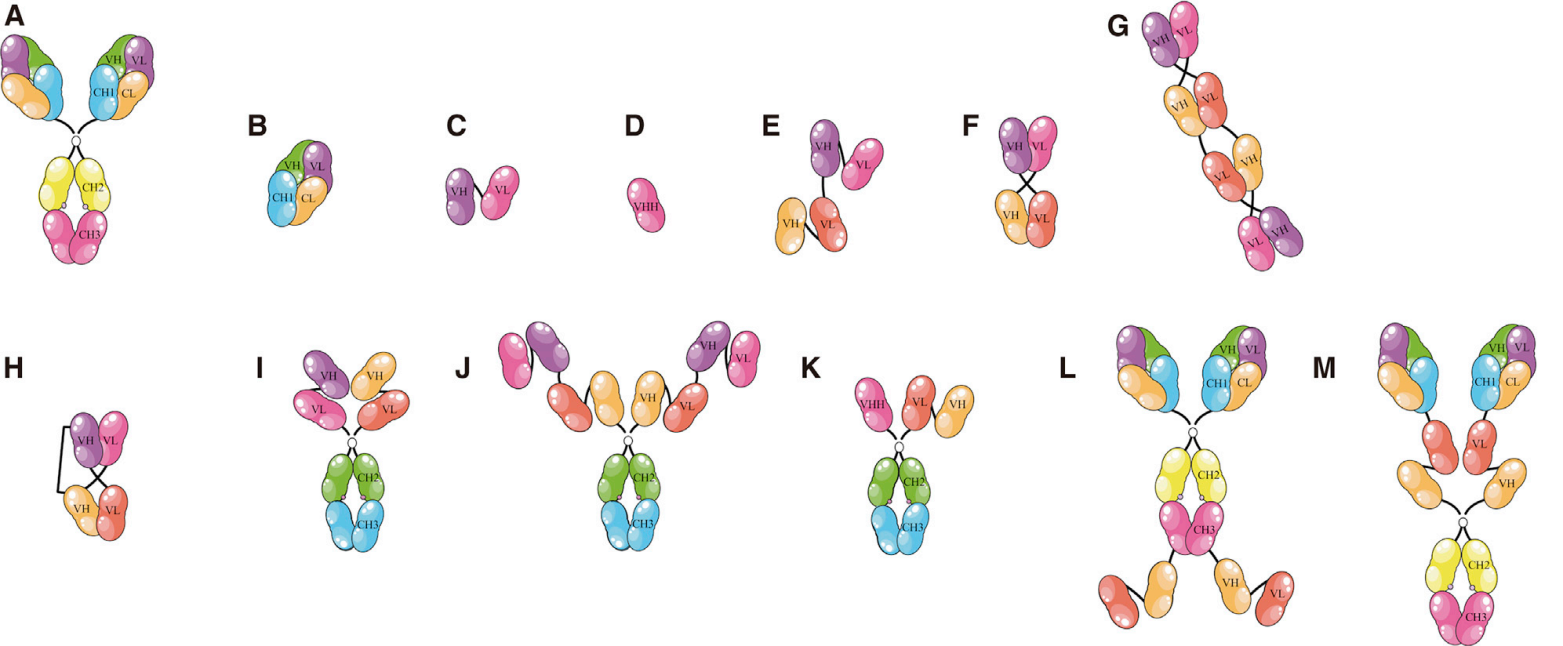
The Schematic Representation of Various scFv-Based bsAb Formats These bsAbs either employing immune cells, such as T cells and NK cells, and bringing them close to the target cell or targeting two vital markers on the cell(s) could exert their therapeutic functions in a range of disorders, such as cancers, autoimmune diseases, and infectious diseases. (A) Whole antibody, (B) antigen-binding fragment (Fab), (C) single-chain variable fragment (scFv), (D) single-domain antibody (e.g., VHH), (E) tandem scFv (e.g., bispecific T cell engager), (F) diabody, (G) tandem antibody (TandAb), (H) dual-affinity retargeting (DART), (I) scFv_2_-Fc, (J) scFv_2_-scFv_2_-Fc, (K) scFv-VHH-Fc, (L) anti-IGF-1R IgG-scFv_2_, and (M) Fab_2_-scFv_2_-Fc (e.g., BiS4aPa) are shown.

Antibodies encompass two identical heavy chains (∼55 kDa) and two identical light chains (∼25 kDa) linked by disulfide and noncovalent bonds, representing a Y-shaped molecule with a molecular weight of 150 kDa (Figure 1A).^3^ Both light and heavy chains are divided into the variable (V) and constant (C) regions. The light chain has one variable domain (VL) and one constant domain (CL), and the heavy chain has one variable domain (VH) and at least three constant domains (CH1-CH2-CH3). Based on function, the structure of these glycoproteins is divided into two parts, including the antigen binding sites and the Fc region. The N terminus parts of both light and heavy chains form the former, and the C terminus domains of the two heavy chains form the latter.^3^ Each antibody has two Fab (fragment antigen binding) arms, consisting of the light chains and the VH and CH1 domains of the heavy chains, carrying the antigen-binding sites (each 50 kDa; Figure 1B). It is noteworthy that the Fc region is involved in antibody effector functions, including antibody-dependent-cell-mediated cytotoxicity (ADCC), antibody-dependent-cellular phagocytosis (ADCP), and complement-dependent cytotoxicity (CDC).^3^

Valency, specificity, affinity, and avidity are four underlying definitions with great effects on the functional properties of antibodies.^5^ The valency is the number of antigen-binding sites on an antibody molecule.^6^ The ability to discriminate a particular epitope from other epitopes by an antibody is defined as the specificity.^3^ Affinity (intrinsic affinity) is defined as the strength of the interaction between an epitope on the antigen and a paratope on the antibody, measured by the equilibrium dissociation constant (K_D_).^5^, ^6^ Avidity (functional affinity) describes the combined strength of multiple antigen-antibody interactions.^5^, ^6^

Thanks to genetic engineering and the emergence of methods, such as phage display, a group of constructs, including the Fab, single-chain variable fragment (scFv) (Figure 1C), and single-domain antibody (sdAb) (Figure 1D), are designed and developed.^7^, ^8^ The scFv consists of two variable domains connected by a flexible linker that is generally the (G_4_S)_3_ sequence (25 kDa; VL_mAbA_-VH_mAbA_; derived from the parental mAbA).^9^ The sdAb only contains a single variable domain (12–15 kDa), such as nanobodies (VHHs) derived from camelid heavy-chain antibodies (HCAbs).^10^ Following the clinical and commercial success of mAbs and antibody fragments, attention was drawn to the development of products with multiple capabilities, leading to the next generation of antibodies, such as bispecific antibodies (bsAbs).^11^ Chemical conjugation of two different mAbs, generation of a quadroma cell line (a hybrid hybridoma secreting a bsAb), and genetic engineering are the most routine methods used to produce the bsAbs.^12^ Particularly, the latter is the strategy thoroughly employed to generate fully human bsAbs, owing to the immunogenicity of mouse-origin antibodies.^13^

The dual targeting is a particular concept linked with bsAbs, enabling them to target two different antigens on two different cells, two different antigens on the same cell, or two different epitopes on the same antigen.^13^, ^14^ Compared with conventional mAbs, the bsAbs can bring immune effector cells, such as T cells and natural killer (NK) cells, alongside the tumor cell, resulting in the efficient tumor cell killing.^15^, ^16^ Simultaneous binding of bsAbs to two different antigens on the surface of the target cell causes the enhanced binding specificity and the concurrent blockade of two different pathways involved in the disease pathogenesis.^17^, ^18^, ^19^ As single molecules, bsAbs have a low production cost in comparison with those of the two mAbs needed for combination therapy.^20^

The bsAbs are classified into two groups of immunoglobulin G (IgG)-like and non-IgG-like molecules based on containing the Fc region or not.^12^, ^20^ The former ones, encompassing the Fc region, have effector functions (ADCC, ADCP, and CDC), easy purification, and a prolonged serum half-life due to their size above the renal clearance threshold and the neonatal Fc receptor (FcRn)-mediated recycling.^9^, ^21^ On the contrary, although the non-IgG-like molecules, devoid of the Fc region, have a short half-life due to their low molecular weight, they benefit from superior tumor penetration, better epitope accessibility, less immunogenicity, and less complicated production compared with the IgG-like molecules.^9^, ^21^ Therefore, different strategies are employed to improve the serum half-life of these molecules, including fusion with human serum albumin (HSA) or the Fc part of an IgG molecule (IgG-like molecules), and polyethylene glycolylation.^22^, ^23^ This group includes tandem scFvs, diabody (single-chain and tandem diabodies), dual-affinity retargeting molecules (DARTs), etc.

### The scFv-Based bsAbs

The scFv is one of the attractive fragments used as the building block of most bsAbs.^11^ This molecule has advantages, such as preservation of the binding activity of the parental antibody, efficient expression in a wide range of hosts (e.g., bacteria and mammalian cells), and great tumor tissue penetration.^17^, ^24^, ^25^ On the other hand, the small size of the scFv causes a short *in vivo* half-life owing to the rapid blood clearance and poor retention time in the target tissues.^9^, ^24^ According to these limitations, tandem scFv molecules (ta-scFvs) consisting of two scFvs fused by a peptide linker (VL_mAbA_-VH_mAbA_–VL_mAbB_-VH_mAbB_) were generated (50–60 kDa; Figure 1E).^11^ These bivalent molecules contain one binding site for each individual antigen.^21^ The long linker between the two scFvs can enhance the flexibility of antigen binding sites of the ta-scFv, leading to better binding to two different targets.^20^ The bispecific T cell engager (BiTE) is one type of ta-scFv that consists of two scFvs, one binds to CD3 on T cells and the second one binds to an antigen on the tumor cell.^11^, ^27^ Concurrent binding of the BiTE to the T cell and the tumor cell triggers a cascade of events, including T cells activation, the release of cytokines engaging other immune cells, and the secretion of perforin and granzyme B, leading to the tumor cell apoptosis.^28^, ^29^, ^30^ Nevertheless, BiTEs also have a short half-life; hence, the continuous intravenous (cIV) infusion is required to provide the optimal serum concentration.^31^

A diabody consists of two polypeptide chains, one of which contains the VH of the antibody A connected with the VL of the antibody B, and the other one contains the VH of the antibody B connected with the VL of the antibody A (VH_mAbA_-VL_mAbB_/VH_mAbB_-VL_mAbA_; Figure 1F).^9^, ^11^ Furthermore, this heterodimeric molecule can be designed with a different configuration (VL_mAbA_-VH_mAbB_/VL_mAbB_-VH_mAbA_).^21^ Each variable domain is connected to another one by a short peptide linker (five amino acids).^21^ Similar to BiTEs, diabodies have two different antigen-binding sites.^21^ Owing to the incorrect dimers generated in the cell and instability of diabodies, different formats of diabody, including double-chain diabody and single-chain diabody, are developed to enhance the stability of the construct.^9^, ^21^ In the former, a disulfide bond is introduced between the domains of one chain, and in the latter, two polypeptide chains are fused by a flexible peptide linker (15 amino acids).^21^ Based on the single-chain diabody format, a dimeric molecule with four binding sites, the tandem diabody (TandAb), is generated (Figure 1G).^12^, ^21^ The TandAb molecules with molecular weight of about 114 kDa and bivalent binding for each antigen exhibit a prolonged half-life and higher binding affinity to the targets compared with ta-scFvs and diabodies.^9^ The other diabody-based bsAb is a DART containing two polypeptide chains (VL_mAbA_-VH_mAbB_/VL_mAbB_-VH_mAbA_) linked by a disulfide bond, causing more stability and easy manufacturability (Figure 1H).^21^, ^27^, ^32^

### The scFv-Based bsAbs in Preclinical Development

*In vivo* production and *in situ* secretion of bsAbs by genetically engineered cells is one of the attractive strategies employed to circumvent problems of short half-life, low penetration into tumor sites, production costs, and severe adverse events, such as cytokine release syndrome observed in patients after a systemic administration of bsAbs.^33^, ^34^ In this regard, different studies demonstrated that genetically modified human primary peripheral blood lymphocytes or endothelial cells could secrete a functionally active carcinoembryonic antigen (CEA)×CD3 diabody redirecting T cells toward CEA-positive tumor cells, resulting in significant tumor growth inhibition *in vivo*.^33^, ^34^, ^35^ Furthermore, two different bsAb formats, including a ta-scFv and a diabody, constructed with variable domains of mAbs targeting CEA and CD3, were compared based on their *in vivo* secretion and ability to activate T cells.^36^ The results unveiled that, although both proteins were secreted from engineered human cells with similar yields, the ta-scFv had a superior tendency to form aggregates resulting in TCR/CD3 cross-linking and thereby target-independent T cell activation.^36^ Together, it was proposed that, although the noncovalent connection between the two chains of diabody might lead to decreased binding capacity (based on the amount of assembled diabody), the diabody format is preferred due to the lack of aggregation leading to adverse events.^36^

To increase the production of functional assembled diabodies and reduce free diabody chain that might disrupt the binding of the assembled diabody to the antigen, a self-cleaving 2A peptide derived from a foot-and-mouth disease virus was incorporated into the two-chain CEA×CD3 diabody gene.^37^ The co-translational “cleavage” of diabody chains by the 2A self-cleaving peptide and subsequently augmented production and secretion of assembled diabodies by genetically engineered human cells led to the improved binding of the bsAb to the antigen (CEA) and enhanced T cell cytotoxicity against CEA-positive tumor cells.^37^

To augment the antitumor activity of ta-scFv bsAbs, Ahmed et al.^38^ constructed an engineered ta-scFv molecule composing of a scFv antibody targeting the carbohydrate epitope on disialoganglioside antigen GD2 (GD2) and a scFv antibody targeting CD3 on T cells. This molecule contained a human hepatocyte nuclear factor 1α (HNF1α) dimerization domain (HDD) added to the C terminus of the molecule that was distal to the GD2-specific scFv placed at the N terminus.^38^ As the CD3-specific scFvs located proximal to the HDD, forming tightly anti-parallel helices, were sterically restricted, monovalency to CD3 was maintained in the HDD-dimeric bsAb. This event prohibited excessive cytokine release. Furthermore, the dimeric GD2×CD3 ta-scFv (∼118 kDa) exhibited enhanced avidity to GD2, increased T cell cytotoxicity against GD2-positive tumor cells, prolonged half-life, and significant antitumor activity in mouse xenograft models.^38^

Despite the significant reduction in AIDS-related mortality since 2004, HIV-1 is still one of the major public health challenges, with 36.9 million infected people (1.8 million children <15 years) and 940,000 AIDS-related deaths worldwide in 2017.^39^ Among various strategies employed up to now to eradicate HIV-1,^40^, ^41^ passive immunotherapy with potent and broadly neutralizing antibodies (bNAbs) seems as an effective strategy to prevent or cure the HIV-1 infection.^41^, ^42^ Due to the inherent plasticity of HIV-1, it is demonstrated that the employment of two bNAbs targeting non-overlapping epitopes or bsAbs simultaneously binding to targets involved in the HIV-1 infection is more efficacious than using a single bNAb.^43^ Indeed, the latter leads to the development of a group of bsAbs with different formats.^19^, ^42^, ^43^, ^44^, ^45^, ^46^ In this regard, Mouquet et al.^19^ generated three scFv_2_-Fc IgG-like molecules (immunoadhesins) consisting of one scFv against a non-neutralizing epitope on gp41 and the other derived from one of the three different antibodies targeting neutralizing epitopes on gp120 (Figure 1I). To promote the heterodimer formation, the “knobs-into-holes” strategy was used.^19^ Indeed, the “knobs-into-holes” strategy is the introduction of a “knob,” substituting a small amino acid with a larger one in the CH3 domain of one heavy chain and a “hole,” substituting a large amino acid with a smaller one in the CH3 domain of the other heavy chain.^45^, ^47^, ^48^ Simultaneous binding of anti-gp41/120 bsAbs to both antigens led to increased viral neutralization in comparison with the parental anti-gp120 IgG antibodies. Indeed, their results demonstrated that the enhanced HIV-1 neutralization resulted from the increased avidity of the anti-gp41/120 bsAb.^19^

In order to cover the cell surface of CD4^+^ T cells with bsAbs (blockade of virus entry) and boost their binding affinity for targets,^19^, ^45^ Wu et al.^44^ generated a single-gene-encoded tandem bispecific immunoadhesin molecule (BiIA-SG) containing two scFvs for gp120 and two scFvs for CD4 (Figure 1J). It was constructed with the scFv of an anti-gp120 antibody (PGT128) and the scFv of an anti-CD4 antibody (Hu5A8) fused to the CH2-CH3 domains of human IgG in tandem (Figure 1J). Due to the two gp120-specific scFvs and the subsequent improved binding to gp120, BiIA-SG exhibited significantly enhanced breadth and potency (IC_50_ value of 0.073 μg/mL against 124 HIV-1 strains). Moreover, the administration of a single dose of BiIA-SG led to sterile protection against divergent HIV-1 challenges in humanized mice. In a short-term treatment setting (for 3 weeks), BiIA-SG plus combination antiretroviral therapy (ART) could delay viral rebound. Consequently, they reported that the administration of a single high dose of the adeno-associated virus-transferred BiIA-SG gene resulted in the elevated half-life of BiIA-SG and thereby eliminating infected cells in humanized mice.^44^

To improve the T-cell-mediated eradication of HIV-1-infected cells, Sung et al.^46^ designed two HIV×CD3 DARTs containing an anti-HIV-1 binding arm (derived from mAb A32 specific to gp120 or mAb 7B2 specific to gp41) linked to an anti-CD3 binding arm. These molecules comprised two polypeptide chains, one constructed with the VL of the anti-CD3 antibody combined with the VH of the anti-HIV antibody and the other one constructed with the VL of the anti-HIV antibody combined with the VH of the anti-CD3.^46^ Two HIV×CD3 DARTs could redirect cytolytic T cells to kill a panel of cells expressing HIV-1 envelope (infected CD4^+^ T cells). In an autologous system, the DARTs could recruit CD8^+^ T cells isolated from patients with HIV-1 infection receiving suppressive ART to eradicate infected CD4^+^ T cells.^46^

CD47/SIRPa signaling (“don’t eat me” signal) is one of the pathways harnessed by tumor cells to escape from phagocytosis by immune cells.^49^, ^50^, ^51^ CD47 is a checkpoint receptor overexpressed in different hematological (e.g., acute myelogenous leukemia [AML] and non-Hodgkin lymphoma [NHL]) and solid malignancies (e.g., colon, bladder, and brain).^49^, ^50^ To interfere with the CD47/SIRPa interaction, different therapeutic agents are developed, including anti-CD47 mAbs, SIRPa blocking agents, SIRPa-Fc fusion proteins, and bsAbs targeting CD47 and the tumor target. Although anti-CD47 mAbs (Hu5F9-G4 and CC-90002) and a SIRPa-Fc fusion protein (TTI-621) could recently enter phase I clinical trials, it is stated that the ubiquitous expression of CD47, as well as the Fc region, likely affects the therapeutic efficacy, safety, and tolerability of these agents in patients with solid tumors and blood malignancies.^49^ In this regard, Celgene halted a phase I, open-label dose-escalation and expansion trial of CC-90002 in patients with AML and myelodysplastic syndrome (MDS) (ClinicalTrials.gov: NCT02641002). In fact, the broad expression of CD47 throughout the human body causes toxicity and adverse events in patients receiving CD47 blocking agents and creates an antigen sink that avoids agents from reaching the target site.^51^ To circumvent these limitations, bsAbs with low affinity for CD47 but high affinity for the tumor antigen (e.g., CD19 and CD20) were designed.^50^ In this regard, two bsAbs were constructed, including a CD47×CD20 dual variable-domain Ig (DVD-Ig) molecule targeting CD47 and CD20 and a kλ-body targeting CD47 and CD19.^49^, ^50^ The latter was a fully human IgG1 with two different light chains, including a kappa light chain binding to CD47 and a lambda light chain binding to CD19.^49^ Although both bsAbs could efficiently kill tumor cells, the binding of their Fc part to Fc receptors (FcRs) on phagocytes might lead to the toxicity and decreased efficacy *in vivo* (low free antibodies can reach the targets on tumor cells).^49^, ^50^, ^51^ Therefore, van Bommel et al.^51^ constructed a novel ta-scFv bsAb (RTX-CD47) comprising two scFvs derived from rituximab and from an anti-CD47 mAb. Due to the monovalency of RTX-CD47 to each antigen, it was essential that RTX-CD47 initially bound to CD20, promoting binding to CD47, and thereby inhibiting CD47/SIRPa signaling. In fact, CD20-restricted blocking of CD47/SIRPa interaction through RTX-CD47 prevented toxicity resulting from blocking of CD47 on normal cells. Due to the lack of the Fc part, RTX-CD47 was devoid of off-target activation of phagocytic cells. *In vitro*, RTX-CD47 could potentiate phagocytic removal of CD20^+^/CD47^+^ cells by human phagocytes and synergistically augmented phagocytosis of malignant B cells when combined with obinutuzumab (anti-CD20 mAb), daratumumab (anti-CD38 mAb), or alemtuzumab (anti-CD52 mAb).^51^

The epidermal growth factor receptor variant III (EGFRvIII) is a truncated receptor resulted from the in-frame deletion of 801 base pairs of exons 2–7 in the EGFR gene.^52^ Due to this deletion, EGFRvIII is constitutively active in a ligand-independent fashion, and its aberrant signaling is associated with glioma growth and progression.^53^ Two different groups individually developed TandAb and BiTE molecules targeting EGFRvIII and CD3.^54^, ^55^ The particular feature of both bsAbs is their EGFRvIII-specific scFvs. By the phage display technology and fully human antibody libraries, Ellwanger et al.^54^ isolated a low-affinity scFv antibody, Li3G30, identifying EGFRvIII. Following affinity maturation, high-affinity scFvs (K_D_s in the range of 0.25–6.50 nM) were used to construct TandAbs that could completely discriminate EGFRvIII from the wild-type EGFR. *In vitro*, highly potent EGFRvIII×CD3 TandAbs by engaging T cells could kill different EGFRvIII-positive cells with EC_50_ values ranging from 1 to 10 pM, without any cytotoxic effects on EGFR-positive cells or EGFRvIII-negative cells. Furthermore, they demonstrated that EGFRvIII×CD3 TandAbs with high-affinity binding to CD3 did not activate T cells co-cultured with EGFRvIII-negative cells. In a dose-dependent manner, the TandAbs could inhibit the tumor growth in a tumor xenograft model derived from the EGFRvIII-positive cell line.^54^

In another study, Gedeon et al.^55^ generated a library of ta-scFvs (BiTEs) consisting of variable domains derived from human anti-EGFRvIII (mAb clone 139) and human anti-CD3 (foralumab; 28F11-AE; NI-0401) antibodies. Following multiple screening and assessments, one fully human bsAb, EGFRvIII×CD3 BiTE molecule, was selected for further evaluation. By recruiting human T cells, the selected BiTE molecule potently and selectively lysed EGFRvIII-positive cells (malignant glioma cell lines and patients-derived glioma cells). Both in orthotopic and subcutaneous glioma models, the intravenous (i.v.) administration of EGFRvIII×CD3 BiTE molecule led to the regression of established tumors and prolonged survival in patient-derived glioma xenografts and could cure established, highly aggressive, syngeneic glioma.^55^

Overexpression of human epidermal growth factor receptor 2 (HER2) (two million molecules on the surface of a tumor cell) in 20%–30% of breast cancers and its correlation with disease progression and poor prognosis in patients result in the design and development of a bunch of HER2-targeted therapeutic agents, including trastuzumab (4D5; Herceptin), ado-trastuzumab emtansine (T-DM1; Kadcyla), pertuzumab (2C4; Omnitarg), and lapatinib (Tykerb/Tyverb).^10^, ^56^, ^57^, ^58^, ^59^ Nanobodies (VHHs) are sdAbs derived from functional HCAbs.^10^, ^60^, ^61^ The small size (15 kDa) and single domain entity endow nanobodies particular features, including binding ability to “hard to reach” epitopes, high-affinity binding, high stability, easy cloning, and easy expression and purification from *Escherichia coli*.^10^, ^60^, ^61^ Hence, Xing et al.^60^ constructed an IgG-like bsAb with the mutated Fc, BiHC (a bispecific HER2-CD3 antibody), comprising an anti-HER2 nanobody and an anti-CD3 scFv (derived from a humanized UCHT1 antibody) linked to CH2 and CH3 domains (Figure 1K). Moreover, they employed the knobs-into-holes strategy to facilitate the Fc heterodimerization.^60^ The BiHC exhibited cytotoxicity against HER2-positive cell lines in a target- and dose-dependent fashion. This molecule could also effectively inhibit the tumor growth in a cell-line-derived xenograft mouse model.^60^ The scFv-based bsAbs in preclinical development are summarized in Table 1.

**Table 1:** The scFv-Based bsAbs in Preclinical Development

bsAb	Format	Biological Activity	References
Anti-CEA×anti-CD3	diabody	redirecting T cells to CEA-positive tumor cells	36, 37
Anti-GD2×anti-CD3	BiTE	redirecting T cells to GD2-positive tumor cells	^38^
Anti-gp41×anti-gp120	scFv_2_-Fc	enhanced HIV-1 neutralization	^19^
Anti-gp120×anti-CD4 (BiIA-SG)	scFv_2_-scFv_2_-Fc	elimination of HIV-1-infected cells	^44^
Anti-gp120×anti-CD3	DART	redirecting T cells to kill a panel of Env-expressing cells	^46^
Anti-gp41×anti-CD3			
Anti-CD47×anti-CD20	ta-scFv	inhibition of “don’t eat me signaling” and phagocytic removal of CD20^+^/CD47^+^ cells	^51^
Anti-EGFRvIII×anti-CD3	TandAb	redirecting T cells to EGFRvIII-positive cells	^54^
Anti-EGFRvIII×anti-CD3	BiTE	redirecting T cells to EGFRvIII-positive cells	^55^
Anti-HER2×anti-CD3 (BiHC)	scFv-VHH-Fc	redirecting T cells to HER2-positive tumor cells	^60^

BiHC, bispecific HER2-CD3 antibody; BiIA-SG, single-gene-encoded tandem bispecific immunoadhesin; BiTE, bispecific T cell engager; CEA, carcinoembryonic antigen; DART, dual-affinity retargeting molecule; EGFRvIII, epidermal growth factor receptor variant III; Env, envelope; GD2, disialoganglioside GD2; gp41, glycoprotein 41; gp120, glycoprotein 120; HER2, human epidermal growth factor receptor 2; scFv, single-chain fragment variable; TandAb, tandem antibody; ta-scFv, tandem scFv; VHH, the variable domain of camel heavy-chain antibodies.

### The scFv-Based bsAbs in Clinical Trials

Overexpression in a wide range of carcinomas, such as lung, breast, colorectal, gastric, pancreas, and ovarian cancers; restricted expression in normal epithelial cells; and involvement in cancer progression make the epithelial cell adhesion molecule (EpCAM) an ideal target for immunotherapy-based approaches, including mAb therapy and adoptive T cell therapy.^62^, ^63^, ^64^ Catumaxomab (Removab) is the first bispecific trifunctional antibody composed of one mouse light and heavy chains specific for human EpCAM and one rat light and heavy chains specific for human CD3, as well as the Fc region preferentially binding to Fc activation receptors (Fc gamma receptor [FcγR]I, FcγRIIa, and FcγRIII) on macrophage, NK, and dendritic cells.^65^ Therefore, catumaxomab by harnessing immune effector cells potentiates the immune system of patients, leading to the eradication of tumor cells through cytolytic activation of T cells, phagocytosis, and ADCC.^65^ Cytokine-release-related events and hepatotoxicity are the common adverse events reported with catumaxomab.^65^ Catumaxomab was approved in 2009 by the European Medicines Agency (EMA) to treat patients with malignant ascites and withdrawn in 2017 in the European Union market due to commercial reasons.^65^, ^66^

MT110 (also known as AMG 110 or solitomab) is an EpCAM×CD3 BiTE molecule.^16^, ^62^ MT110 benefits from an EpCAM-specific scFv with a moderate affinity isolated by the phage display technology.^16^ Because identification of EpCAM on normal tissues by high-affinity anti-EpCAM mAbs (*K*_D_ of 1 nM) leads to severe adverse events, such as acute pancreatitis in patients, the moderate affinity of MT110 for EpCAM (ranging from 10 to 100 nM) enables it to discriminate EpCAM-overexpressing tumor cells from normal epithelial cells with low levels of accessible EpCAM.^16^ It also exhibited good manufacturability and lower immunogenicity (using a de-immunized anti-CD3 scFv) compared with MT102, another anti-EpCAM BiTE molecule.^16^ *In vitro*, MT110 exhibited a potent activity in killing EpCAM-overexpressing tumor cells by redirected unstimulated human peripheral T cells (CD4^+^ and CD8^+^).^16^, ^62^ MT110 was efficacious in three *in vivo* models (tumor growth inhibition and elimination of established tumors).^16^ Consequently, a multicenter phase I study assessed the tolerability, pharmacokinetics (PKs), pharmacodynamics (PDs), and antitumor activity of MT110 in patients with relapsed and/or refractory (R/R) EpCAM-positive solid tumors not amenable to standard therapy (ClinicalTrials.gov: NCT00635596). Patients received MT110 (1–96 μg/day) as cIV infusion (due to a half-life of 4.5 h) for ≥28 days. The maximum tolerated dose (MTD) was reported 24 μg/day. Despite the antitumor effects in two patients with ovarian cancer, dose-limiting toxicities (DLTs), such as severe diarrhea and elevated liver enzymes, impeded dose escalation to therapeutic levels.^63^ Conclusively, they attributed these adverse events to the EpCAM expression in the bile duct and gastrointestinal (GI) epithelial cells and mode of BiTEs action.^63^

Overexpression in tumor cells and the correlation between the elevated serum levels of soluble CEA (sCEA) shedding from tumor cells with disease progression made CEA as one of the most important biomarkers in cancers, such as colorectal cancer (CRC).^67^, ^68^, ^69^ On the other hand, sCEA may interfere with immunotherapy approaches targeting membrane-bound CEA.^67^, ^68^, ^69^ To that end, Lutterbuese et al.^67^ constructed a series of BiTE antibody constructs, consisting of CD3- and CEA-specific scFvs that their subsets were not competitively inhibited by sCEA. They assessed the ability of CEA×CD3 BiTE molecules in redirecting of T cells to lyse CEA-positive tumor cells, demonstrating the specificity and potent cytotoxic activity of the molecules in both *in vitro* and *in vivo* assays.^67^ In another study, Osada et al.^68^ reported that the low concentration (0.1–1 ng/mL) of MEDI-565 (also known as AMG 211), developed from the mentioned constructs, could engage patients’ T cells to kill CEA-positive CRC specimens derived from patients that previously received conventional chemotherapy. Moreover, they showed that both CD4^+^ and CD8^+^ T cells were involved in T-cell-mediated killing of CEA-positive tumor cells in agreement with previous data published by Kischel et al.^68^, ^69^, ^70^, ^71^ MEDI-565 is evaluated in three phase I studies in patients with GI adenocarcinomas (ClinicalTrials.gov: NCT01284231, NCT02291614, and NCT02760199). The results from the first one, an open-label, dose-escalation study, showed DLTs, such as hypoxia (in two patients) and cytokine release syndrome (in one patient), linear PK, a short half-life as expected, no objective responses, and stable disease in 11 patients (28%) receiving 5 mg MEDI-565 as an intermittent 3-h infusion for 5 consecutive days (ClinicalTrials.gov: NCT01284231).^71^ The second study assessed the safety, PK, PD, and antitumor activity of MEDI-565 in patients with R/R GI adenocarcinoma (ClinicalTrials.gov: NCT02291614). In order to reduce adverse events and achieve a better therapeutic index, patients received a cIV administration of MEDI-565 (0.2–12.8 mg/day).^72^ More than 20% of the patients showed adverse events related to the GI system, and anti-MEDI-565 antibodies were observed in all patients receiving doses of more than 3.2 mg of MEDI-565. Conclusively, a therapeutic window was not defined for MEDI-565 due to immunogenicity limiting adequate exposure for objective responses.^72^ The third study evaluated the usefulness of Zirconium-89-labeled MEDI-565 positron-emission tomography (PET) scan in patients with R/R GI adenocarcinoma before and during treatment with MEDI-565 (ClinicalTrials.gov: NCT02760199). The former could help determine the uptake and distribution of radiolabeled MEDI-565 in primary and metastatic tumor lesions and normal organs, and the latter could help assess the impact of prolonged MEDI-565 exposure on tumor and tissue uptake. The results of the last study are not yet published.

Second to lung cancer, prostate cancer is the most life-threatening cancer among American males, with an estimate of 29,430 deaths in 2018.^73^, ^74^ The selection of appropriate targets is a critical step in the development of BiTEs; prostate-specific membrane antigen (PSMA) is one of the ideal candidates, which is highly expressed in prostate adenocarcinoma and plays an underlying role in the progression of prostate cancer.^75^, ^76^ Despite the different PSMA-specific bsAbs developed up to now, the particular features of BAY2010112 (also known as MT111, AMG 212, or pasotuxizumab), an anti-PSMA BiTE antibody designed by Friedrich et al.^73^ distinguishes it from other previous constructs, such as diabodies constructed by Buhler et al.^77^, ^78^ and Fortmuller et al.^79^ These diabodies have drawbacks, including the murine origin of their components and the anti-CD3 scFv derived from OKT-3 lacking the ability to react with cynomolgus monkey T cells.^73^, ^77^, ^78^, ^79^, ^80^ On the contrary, the amino acid sequence of BAY2010112 is very close to the variable gene segments of human Ig, leading to less immunogenicity.^73^ BAY2010112 consists of scFvs that can react with both human and monkey PSMA and CD3 antigens, providing a nonclinical safety evaluation of BAY2010112 in the cynomolgus monkey. In a target-dependent fashion, BAY2010112 could induce T-cell-mediated cytolysis of prostate cancer cell lines with EC_50_ values ranging from 0.1 to 4 ng/mL, depending on the cell line.^73^ This is while, in the two abovementioned studies, the PSMA×CD3 diabodies showed EC_50_ values of 1.4^79^ and 15 ng/mL.^77^, ^78^ In addition to remarkable inhibition of tumor formation similar to the mentioned diabodies, BAY2010112 could cause complete remissions in mice with established prostate cancer xenografts.^73^ BAY2010112 is now investigated in a phase I, open-label, dose-escalation study to determine its MTD, safety, tolerability, and PK in patients with castration-resistant prostate cancer (ClinicalTrials.gov: NCT01723475).

AML is one of the two most common types of leukemia in adults with 10,670 deaths in the US in 2018.^74^, ^81^ As AML progresses quickly, patients need prompt treatment, including chemotherapy, allogeneic stem cell transplantation, and targeted therapy.^82^, ^83^ Although mAb-based immunotherapy is a treatment option for patients with AML, it needs AML-specific targets to obtain promising results.^84^ The broad expression of CD33 on AML blasts in a large population of AML patients (∼90%), in addition to no expression in normal hemopoietic stem cells, qualify CD33 as a suitable target for AML immunotherapy.^85^, ^86^ Although antibody-based immunotherapies have great impacts on the treatment of B cell malignancies, AML has not significantly benefited until now, especially after withdrawal of gemtuzumab ozogamicin (an anti-CD33 antibody-drug conjugate) from the market of the US and no further clinical development of lintuzumab (an anti-CD33 mAb) and vadastuximab talirine (an anti-CD33 antibody-drug conjugate).^84^, ^87^ Among different antibody-related therapeutics (e.g., camidanlumab tesirine and actinium-225-lintuzumab) in AML clinical trials, AMG 330 is a novel CD33×CD3 BiTE molecule currently in a phase I clinical trial to evaluate its therapeutic efficacy in patients with R/R AML (ClinicalTrials.gov: NCT02520427).^84^, ^85^, ^87^, ^88^ Soluble CD33 (sCD33), which inhibits binding of AMG 330 to its target on AML cells, and *de novo* expression of CD33 on activated T cells, turning them to target cells for AMG 330, are the two critical points affecting the efficacy of AMG 330.^87^ Nonetheless, Friedrich et al.^87^ reported that AMG-330-mediated lysis was scarcely influenced by sCD33 at concentrations up to 100 ng/mL, and CD33-positive T cells activated by blinatumomab only constituted 6% of all T cells. *In vitro*, AMG-330-redirected T cells could potently lyse AML cell lines expressing different levels of CD33 (14,400 and 56,700 molecules/cell) with EC_50_ values ranging 18–149 pg/mL.^87^ In an autologous system, AMG 330 could recruit T cells derived from patients with AML to deplete AML blasts.^89^ According to the ability of AMG 330 to interact with CD33 and CD3 of both human and nonhuman primates, AMG 330 could redirect autologous T cells against CD33-positive cells isolated from monkey bone marrow samples in an *ex vivo* assay.^87^ Furthermore, AMG 330 exhibited the antitumor activity *in vivo*, leading to the elevated survival in a mouse xenograft model of leukemia.^87^ In a phase I dose-escalation study to evaluate the safety, tolerability, PK, PD, and efficacy of AMG 330 in patients with R/R AML, AMG 330 was administrated as a cIV infusion at doses up to 480 μg/day (ClinicalTrials.gov: NCT02520427). Serious treatment-associated adverse events were seen in 15 out of 35 patients. At the dose of 480 μg/day, DLTs were cytokine release syndrome (grade 2) and ventricular fibrillation (grade 4), leading to the reduction of the target dose to 240 μg/day. A complete response was seen in two patients who were on 240 μg/day. In conclusion, they suggested that the BiTE molecules could be suitable therapeutics for the eradication of CD33-positive cells.^90^

Multiple myeloma (MM) is one of the most common hematological malignancies, featured by the unrestrained proliferation of plasma cells in the bone marrow.^91^, ^92^ Aside from advances in the treatment of MM and primary remission, relapse is the main factor threatening patients.^91^ Thus, there is still an unmet medical need for finding therapies with more efficacies in patients not responding to standard treatments.^28^ Among MM-related antigens, B cell maturation antigen (BCMA) has characteristics distinguishing it from others, including overexpression in MM cells, stimulation of MM cells proliferation, and involvement in the upregulation of antiapoptotic proteins and drug resistance.^93^ AMG 420 (formerly BI836909) is a BiTE molecule targeting BCMA and CD3.^28^ *In vitro*, AMG 420 by harnessing and stimulating both CD4^+^ and CD8^+^ T cell subpopulations could specifically lyse BCMA-positive MM cells, in agreement with data from an EpCAM/CD3 bsAb revealing that both T cell subsets play a role in this process.^16^, ^28^ Furthermore, Hipp et al.^28^ demonstrated that the pathological levels of soluble BCMA or a proliferation-inducing ligand (APRIL) could not exert great effects on the activity of AMG 420. In addition to a potent activity *in vivo*, including tumor cells elimination and elevated survival, AMG 420, by retargeting T cells toward MM cells (both autologous and obtained from newly diagnosed and relapsed and refractory MM patients), could exhibit remarkable antitumor activity in *ex vivo* assays. The potential of AMG 420 was also assessed in cynomolgus monkeys, leading to the depletion of BCMA-positive plasma cells in the bone marrow.^28^ The preclinical results motivated Boehringer Ingelheim to launch an open-label, phase I study for measuring the MTD (the primary objective) and the safety, PK, PD, and efficacy (the secondary objectives) of AMG 420 in patients with R/R MM who experienced progression after equal or more than two prior treatment lines (ClinicalTrials.gov: NCT02514239). Based on the results, AMG 420 exhibited a favorable antitumor activity, and the objective response rate was 83% at the dose of 400 μg/day. Treatment-related serious adverse events were cytokine release syndrome and peripheral polyneuropathy observed at the dose of 800 μg/day, although no DLTs were reported up to 400 μg/day. Consequently, PK data revealed that patients who responded to treatment had superior free-drug exposure levels compared to those who did not.^94^

To eliminate EGFRvIII-expressing cells by bsAb-redirected T cells, Amgen launched a phase I, open-label, sequential-dose-escalation study to assess the tolerability, safety, PK, and PD of AMG 596, an EGFRvIII×CD3 BiTE molecule, in patients with glioblastoma expressing mutant EGFRvIII (ClinicalTrials.gov: NCT03296696). This study is conducted with two groups of patients based on disease stage and recurrent disease (the first group) and maintenance treatment after standard of care (SOC) in newly diagnosed disease (the second group).

Although the underlying role of CD19 in the activation and differentiation of B cells is undeniable and its highly conserved expression is observed in most B cell malignancies, such as acute lymphoblastic leukemias (ALL), chronic lymphocytic leukemias, and lymphomas, it is still unknown whether CD19 is directly involved in B cell carcinogenesis or not.^95^ Nevertheless, CD19 is a fascinating substitution for CD20 in the development of therapeutic antibodies used for the treatment of patients with B cell malignancies.^96^ To deplete CD19-positive leukemia and lymphoma cells by retargeted T cells, Reusch et al.^96^ developed a TandAb, AFM11, possessing the same target specificity with blinatumomab. However, the particular structure of AFM11 endows distinct features that discriminate it from BiTE molecules, including a bivalent format and subsequently a greater binding affinity for each target (about 5- and 100-fold higher for CD19 and CD3, respectively) and a molecular weight of about 105 kDa, causing an increased half-life up to 22.9 h.^96^ *In vitro*, AFM11 exhibited a potent cytolytic activity against B cells with little dependency upon the effector: target ratio and induced tumor growth inhibition in a non-obese diabetic (NOD)/severe combined immunodeficiency (SCID) xenograft model in a dose-dependent manner.^96^ In this regard, two phase I studies were launched separately to assess the safety and activity of AFM11 in patients with relapsed or refractory adult B-precursor ALL (ClinicalTrials.gov: NCT02848911) and the ones with R/R CD19-positive B cell NHL (ClinicalTrials.gov: NCT02106091). Nevertheless, both trials were stopped due to serious adverse events, including one death and two life-threatening events in patients with ALL and NHL enrolled in the highest dose cohorts of each study, respectively.^97^

The other anti-CD19 bsAb in the clinical development is a humanized CD19×CD3 DART molecule, designated MGD011 (also known as JNJ64052781 or duvortuxizumab).^98^, ^99^ To shun the target-independent T cell activation, MGD011 was engineered with a mutated Fc domain with no binding ability to FcγRs and complement C1q.^98^ Due to a superior affinity for CD19, compared with CD3, MGD011 initially bound to CD19 on target cells, diminishing the target-independent activation of T cells.^98^ Notably, the engineered MGD011 could bind to the FcRn, increasing its half-life to 161 h. The other key feature of MGD011 was its cross-reaction with CD19 and CD3 antigens in cynomolgus monkeys, providing a preclinical safety evaluation, PK assessment, and dose escalation in this species. In this regard, in addition to the potent antitumor activity *in vitro* and in mice models of lymphoma and leukemia, a weekly administration of MGD011 led to the profound and long-lasting B cells elimination in cynomolgus monkeys.^98^ Furthermore, no cytokine storm (due to monovalent binding) and no infection-related adverse events were observed in monkeys treated with MGD011. Notably, neurotoxicity was not observed in toxicological studies of MGD011 (the predictive value of the cynomolgus monkey to neurologic toxicity associated with CD19×CD3 bsAbs is unknown). Although Janssen, in collaboration with MacroGenics, initiated dosing of MGD011 in a phase I study in patients with relapsed or refractory B cell malignancies (ClinicalTrials.gov: NCT02454270), the former terminated the enrollment of the trial due to clinical concerns for neurotoxicity observed in a number of patients receiving treatment.^100^, ^101^

Overexpression in various tumors, such as ovarian, pancreatic, CRC, and breast cancers, and its connection with invasiveness and poor prognosis in patients with related cancers made P-cadherin a great therapeutic target for cancer therapy.^30^, ^102^ An affinity-optimized scFv with picomolar affinity to P-cadherin, as well as an engineered Fc region in the structure of a DART molecule, led to the generation of PF-06671008 with a prolonged circulation half-life (105.7 h), high stability, high expression, no ADCC activity, and great antitumor activity against P-cadherin-positive cell lines.^30^, ^102^ Of note, in the latter, a significant correlation was observed between cytotoxic activity (EC_50_ values) of PF-06671008-redirected T cells and the surface expression of P-cadherin.^102^ Moreover, PF-06671008, by activating human T cells engrafted to mice, could cause the regression of established tumors in the cell-line- and patient-derived tumor xenograft models.^102^ To assess the safety, PK, and PD of PF-06671008, a phase I dose-escalation study sponsored by Pfizer is being conducted on patients with P-cadherin expressing triple-negative breast cancer, CRC, or non-small-cell lung cancer (ClinicalTrials.gov: NCT02659631).

Overexpression in a large proportion of AML patients has translated CD123 to one of the attractive antigens in the development of CD123-based targeted immunotherapy.^103^, ^104^ The overexpression of CD123, the α chain of interleukin-3 receptor (IL-3R), and thereby IL-3R signaling overactivity are correlated with increased proliferation and enhanced tumor cell viability.^105^ To eliminate CD123-positive AML cells by redirecting T cells, Al-Hussaini et al.^104^ designed a humanized CD123×CD3 bispecific DART molecule. They demonstrated that this molecule, MGD006 (also known as S80880 or flotetuzumab), by simultaneously binding to human CD123 and CD3 induced T-cell-mediated killing of CD123-positive AML cells *in vitro* and *in vivo*.^104^ Similar to MGD011, MGD006 was also able to recognize cynomolgus monkey CD123 and CD3, and its continuous administration for up to 4 weeks demonstrated the well tolerability of MGD006.^104^, ^106^ Based on these promising results, a phase I, open-label, dose-escalation study is ongoing to establish the MTD and to evaluate the safety profile and preliminary anti-leukemic activity of MGD006 (at doses between 3 and 1,000 ng/kg/day) in patients with relapsed or refractory AML or intermediate-2 or high-risk MDS (ClinicalTrials.gov: NCT02152956). The preliminary data revealed that MGD006 was well tolerable, and drug-related adverse events (≥grade 3) observed in 36% of patients were alleviated by the early administration of tocilizumab. MGD006 exhibited anti-leukemic activity in patients at doses of ≥500 ng/kg/day.^107^ Furthermore, MGD006 is currently in a phase II trial study to assess its toxicity profile and antitumor activity in patients with CD123-positive advanced ALL and other hematological malignancies (ClinicalTrials.gov: NCT03739606).

The B-cell antigen receptor (BCR) signaling, triggered by the recognition of an immune complex, in connection with other downstream signaling pathways, leads to B cell proliferation, differentiation, and activation through Ig production.^108^ To prevent the hyperactivation of BCR signaling pathway, the Fc domain of the complex-bound IgG binds to FcγRIIb (CD32B), and as a result, an inhibitory loop is triggered.^109^ To couple the activation signals, such as BCR signaling pathway, involved in autoimmune diseases, with the inhibition one, a DART molecule consisting of humanized variable domains specifically recognizing CD32B and CD79B (CD79B is the Igβ in the signal-transducing part of the BCR complex) was designed by Veri et al.^109^, ^110^ The CD32B×CD79B DART benefits from the two particular characteristics; the first is its CD32B-specific variable domains that show superior affinities for their target compared with the CD79B-specific variable domains, favoring CD32B recognition. The other one is that each component binds to its target in a monovalent manner, making the molecule lack intrinsic activation potential.^109^ They showed that the CD32B×CD79B DART molecule inhibited BCR-induced proliferation and Ig production in activated B cells *in vitro*.^109^, ^111^ The therapeutic effect of the molecule was assessed in a mouse model of collagen-induced arthritis, proving its efficacy in the inhibition of the disease.^109^ To assess the safety, tolerability, and PK of this DART molecule, designated MGD010, as well as its effect on humoral and cell-mediated immune responses in healthy volunteers, a phase I study was conducted on subjects who received a single dose of MGD010 (3 or 10 mg/kg) or placebo, followed by a single-dose administration of hepatitis A vaccine (HAV) (∼50 U; ClinicalTrials.gov: NCT02376036). No serious adverse events were observed in the enrolled subjects. MGD010 exhibited linear PK and dose-dependent binding to peripheral B cells with no B cells elimination but with diminished surface expression of BCR and CD40. In comparison with the placebo group, decreased HAV seroconversion rates with remarkably lower HAV-specific IgG levels were seen in subjects receiving MGD010. Together, the results exhibited that MGD010 had an immunomodulatory activity with a satisfactory safety profile, making it as a suitable candidate for further development as an immunomodulator in patients with autoimmune diseases.^111^, ^112^

CD30 is a member of the tumor necrosis factor receptor superfamily expressed in activated B cells and T cells (both CD4 and CD8 subtypes).^113^ CD30 has emerged as an attractive therapeutic target because of its overexpression in certain malignancies, such as anaplastic large cell lymphoma (ALCL), Hodgkin lymphoma (HL), testicular embryonal carcinoma, on the one hand, and its correlation with cell survival, on the other hand.^113^, ^114^, ^115^, ^116^ Brentuximab vedotin (Adcetris) is an anti-CD30 antibody conjugated with an antineoplastic agent monomethyl auristatin E.^117^ This antibody-drug conjugate was initially approved in 2011 for the treatment of patients with relapsed or refractory^117^ HL and the ones with systemic and primary cutaneous ALCL, not responding to other regimens. Furthermore, in 2018, the US Food and Drug Administration (FDA) approved brentuximab vedotin for the treatment of adult patients with previously untreated stage III or IV classical HL in combination with chemotherapy.^115^ NK cells have always been attractive candidates for cancer immunotherapy due to their high cytotoxic activities. To engage NK cells toward CD30-positive tumor cells, a TandAb molecule, AFM13, with two binding sites for CD16A and two for CD30 was constructed.^15^, ^118^ This TandAb consisted of the affinity-matured human anti-CD16A and murine anti-CD30 variable domains. The former specifically bound to CD16A with no binding to the CD16B isoform, which prevented the elimination of the molecule from the circulation by CD16B^+^ granulocytes.^15^, ^118^ The specific and strong binding to CD30 and CD16A due to the bivalent entity and the resultant reduced K_off_ made AFM13 as a potent and efficacious agent lacking off-target NK cell activation and destroying only CD30-positive tumor cells *in vitro* (with an IC_50_ value of 35.8 nM).^15^, ^118^ Furthermore, no significant cytokine release was observed in toxicological studies either in cynomolgus monkeys or in a phase I clinical study.^119^ A phase I dose-escalation study assessed the safety, tolerability, PK, PD, and antitumor activity of AFM13 (one cycle; once weekly for 4 weeks) in patients with heavily pretreated R/R HL (ClinicalTrials.gov: NCT01221571).^118^ Notably, as no suitable *in vivo* model did exist to exhibit the safety and efficacy of AFM13-mediated NK cell activation against HL cells and there was no previous experience with CD16A-specific antibodies, the dosing schedule of AFM13 was chosen with the focus on the safety of patients rather than efficacy.^118^ Therefore, patients initially received very low doses of AFM13 that subsequently increased by 700 folds.^118^ The infusions of AFM13 at doses ranging from 0.01 to 7 mg/kg showed mild to moderate adverse events.^15^, ^118^ The only DLT in the study was hemolytic anemia in a patient receiving three infusions at 0.5 mg/kg. Due to the murine part of AFM13, anti-drug antibodies were also observed partly in 50% of the patients, and half of the antibodies showed neutralizing potential. Moreover, based on the PK data, the half-life of AFM13 was 19 h.^15^ Despite the activated NK cells and decreased soluble CD30 in the sera of patients, only partial remission was observed in 3 out of 26 evaluable patients (11.5%). The overall response rate and the overall disease control rate in 13 heavily pretreated patients, receiving AFM13 at a dose of ≥1.5 mg/kg, were 23% and 77%, respectively.^118^ Furthermore, AFM13 could be active in brentuximab-vedotin-refractory patients.^15^, ^118^ Taken together, clinical results from this study confirmed the safety, tolerability, and therapeutic activity of AFM13 in patients with R/R HL, providing a proper condition to launch a phase II study for demonstrating the efficacy of AFM13 with an optimized treatment schedule.^118^ In this regard, University of Cologne, in collaboration with Affimed, sponsored an open-labeled, randomized, multicenter, phase II trial in relapsed or refractory HL patients pretreated with both brentuximab vedotin and anti-PD-L1 or anti-PD-1 antibodies (GHSG-AFM13; ClinicalTrials.gov: NCT02321592). Preliminary results reported from the company showed that AFM13 has efficacy as monotherapy in a subset of heavily pretreated subjects.^120^ Zhao et al.^121^ indicated that the combination AFM13 with a PD-1 blockade led to an enhanced antitumor activity due to the cross-talk between NK cells and T cells and suggested that this combination might improve the clinical outcomes in patients with relapsed or refractory HL. Additionally, based on the clinical data demonstrating high response rates in relapsed or refractory HL patients receiving pembrolizumab (Keytruda)^122^, ^123^ and the clinical activity of AFM13 in R/R HL patients in a previous phase I study,^118^ a phase Ib dose-escalation study was launched to assess the safety, tolerability, and preliminary efficacy of a combination of AFM13 and pembrolizumab in such patients not responding to standard treatment, including brentuximab vedotin (KEYNOTE-206; ClinicalTrials.gov: NCT02665650).^124^ Thirty patients were divided into dose-escalation cohorts (cohorts I, II, and III) and the extension cohort (12 and 18 patients, respectively). The most common adverse events were infusion-related reactions (80%).^124^ The overall response rate and complete response rate in the patients treated with the dose and schedule determined for expansion (cohort III and extension cohort) were 87% and 35%, respectively. Taken together, the clinical results of this study demonstrated that the combination of AFM13 and pembrolizumab was a well-tolerated treatment in relapsed or refractory HL patients.^124^ The fourth clinical study is an open-label phase Ib/IIa study evaluating the biological activity of AFM13 in the elimination of CD30-positive tumor cells in patients with relapsed or refractory cutaneous lymphomas (ClinicalTrials.gov: NCT03192202). The preliminary results confirmed that AFM13 as monotherapy had a satisfactory safety profile and therapeutic activity in enrolled patients (the overall response rate of 66%).^125^

The ineffectiveness of HER2-targeted therapy due to the ligand-induced activation of the HER2/HER3 signaling pathway has fortified the hypothesis that the concurrent inhibition of HER2 and HER3 in HER2-overexpressing tumors may be more beneficial than individual targeting of HER2 and HER3.^17^, ^126^ In this regard, based on computation modeling, MM-111, consisting of fully human anti-HER2 scFv (K_D_ of 0.3 nM) and anti-HER3 scFv (K_D_ of 16 nM) linked to the modified HSA, was designed by McDonagh et al.^17^ This molecule, by binding to HER2 on HER2-overexpressing cells and then binding to HER3, prevented the ligand-activated signaling triggered from the HER2/HER3/heregulin complex.^17^ In fact, the antitumor activity of MM-111 was dependent on HER2 overexpression, and its binding to HER3 on cells expressing normal levels of HER2 significantly decreased, indicating the high selectivity and no off-target activity of MM-111.^17^ Of note, the incorporation of HSA between two anti-HER2 and anti-HER3 scFvs led to the prolonged serum half-life of MM-111 in mice (up to 20 h) and cynomolgus monkeys (up to 99 h).^17^ Moreover, the combination of MM-111 and either lapatinib or trastuzumab led to the significant tumor growth inhibition *in vivo*.^17^, ^127^ Based on the promising preclinical data, three phase I open-label studies were conducted to assess the safety, tolerability, and clinical activity of MM-111 as a monotherapy or in combination with trastuzumab, lapatinib, or chemotherapy in patients with advanced HER2-positive cancers (ClinicalTrials.gov: NCT00911898, NCT01097460, and NCT01304784).^127^, ^128^, ^129^, ^130^ In a multi-arm, dose-escalation, phase I study, the safety, tolerability, PK, and antitumor activity of MM-111 in combination with SOC regimens were evaluated in patients with advanced HER2-positive solid tumors (ClinicalTrials.gov: NCT01304784).^126^ The results proved the clinical activity of MM-111 and SOC HER2-directed regimens in patients with an overall clinical benefit rate (defined as the complete response, partial response, and stable disease for at least 4 months) of 55%.^126^, ^131^ Adverse effects were also similar to the adverse effects reported for the regimens alone (e.g., diarrhea, fatigue, decreased appetite, and neutropenia).^126^, ^131^ In order to compare the efficacy of MM-111 plus trastuzumab and paclitaxel with that of trastuzumab and paclitaxel alone, a randomized, open-label, phase II study was carried out in patients with advanced gastric, esophagus, and gastroesophageal junction cancers, terminated due to the low efficacy of the former combination (ClinicalTrials.gov: NCT01774851).

Despite the role of the insulin-like growth factor (IGF) pathway in the proliferation and survival of cancer cells, anti-IGF-1 receptor (IGF-1R) antibodies showed limited clinical efficacy in non-stratified patient populations.^132^ It was indicated that HER3, an underlying driver of phosphatidylinositol 3-kinase (PI3K)/AKT/mTOR signaling, is involved in the resistance to anti-IGF-1R therapies.^132^, ^133^ Fitzgerald et al.^133^ reported that, not only was the IGF-1R signaling pathway co-activated with HER3, but also the IGF-1R inhibition caused the HER3 signaling pathway overactivity.^133^ Hence, the concurrent blockade of IGF-1R and HER3 seems to be compelling because, indeed, the PI3K/AKT/mTOR signaling and related survival pathways are inhibited.^133^ In this regard, MM-141 (also known as istiratumab) was developed by Merrimack Pharmaceuticals, consisting of two anti-HER3 scFvs linked to the C terminus of heavy chains of a fully human anti-IGF-1R antibody (Figure 1L).^133^ MM-141 by simultaneous targeting of IGF-1R and HER3 prevented ligands binding (IGF-1 and IGF-2 to IGF-1R and heregulin to HER3) and decreased the levels of IGF-1R and HER3 on the cell surface, resulting in the inhibition of IGF-1R/ErbB3/PI3K/AKT/mTOR signaling.^132^, ^133^, ^134^ Markedly, in *in vivo* models, MM-141 through the cross-linking ability derived from its tetravalent entity could more decrease the receptor levels compared with the combination of anti-IGF-1R and anti-HER3 antibodies.^133^ Based on preclinical models in which MM-141 could augment docetaxel, gemcitabine, everolimus, and nab-paclitaxel (Abraxane), in an open-label, phase I dose-escalation study, the safety, tolerability, PK, and PD of MM-141 in patients with advanced solid tumors were evaluated in three arms, including arm A (MM-141 alone as a monotherapy), arm B (MM-141 with everolimus), and arm C (MM-141 with Abraxane and gemcitabine; ClinicalTrials.gov: NCT01733004).^132^, ^133^, ^134^ Although, in more than 20% of the patients, nausea, vomiting, decreased appetite, and a headache were observed; patients could tolerate MM-141 (as a monotherapy or in combination with chemotherapy). Notably, the assessment of tumor specimens from treated patients exhibited the low cell surface levels of IGF-1R and HER3, demonstrating receptor internalization due to MM-141 activity. Furthermore, this study revealed that there might be a correlation between the serum levels of IGF-1 in patients before treatment and prolonged antitumor activity.^132^ Based on these results, a randomized, double-blind, phase II study was conducted to compare the efficacy of the combination of MM-141 plus nab-paclitaxel and gemcitabine with that of nab-paclitaxel and gemcitabine alone in metastatic pancreatic cancer patients with high serum levels of free IGF-1 (CARRIE; ClinicalTrials.gov: NCT02399137). The results showed that this combination was not more effective than nab-paclitaxel and gemcitabine alone in enrolled patients.^135^

The antibiotic resistance in the “ESKAPE” group consisting of *Enterococcus faecium*, *Staphylococcus aureus*, *Klebsiella pneumoniae*, *Acinetobacter baumannii*, *Pseudomonas aeruginosa*, and *Enterobacter* species, as well as less tendency of pharmaceutical companies toward developing new antibiotics and toxicity of the existing antibiotics, such as colistin and vancomycin, highlight the need to find novel therapeutic agents reclaiming immunocompromised patients in hospitals.^136^, ^137^ Among ESKAPE pathogens, the large genome coding capacity and intricate regulatory systems have endowed *P. aeruginosa* high adaptation ability to environmental stress.^18^ One of the best strategies to target such pathogens and reduce their resistance is to employ agents with different mechanisms of actions.^18^ In this regard, multifunctional antibodies simultaneously targeting two virulence factors have demonstrated a promising platform in the eradication of pathogens like *P. aeruginosa*. Bs4Ab, a particular platform designed by Bezabeh et al.,^138^ was used to generate a tetravalent bispecific molecule, designated MEDI3902 (formerly, BiS4aPa), consisting of two Fabs against type III secretion protein PcrV and two scFvs against Psl exopolysaccharide, inserted into the hinge domain of a full-length IgG1 mAb (Figure 1M).^18^, ^137^ The virulence factor PcrV involved in the type III secretion system (T3S injectisome) and the persistence factor Psl exopolysaccharide involved in the immune evasion and biofilm formation of *P. aeruginosa* have striking roles in the establishment of acute and persistent infections associated with *P. aeruginosa*.^18^, ^139^ Interestingly, the anti-Psl scFv was inserted into the upper hinge region of MEDI3902 that, by providing an intermediate distance between paratopes, makes an ideal format for concurrent targeting of PcrV and Psl.^18^ Owing to the pervasiveness of PcrV and Psl in global clinical isolates, it was implied that MEDI3902 is able to cover various *P. aeruginosa* strains and related infections.^139^ In this way, BiS4aPa showed remarkable activity against various clinical strains, such as multi-drug-resistant strains in multiple *in vivo* models, including pneumonia, bacteremia, and thermal injury, in both prophylactic and therapeutic regimens.^18^, ^140^ Besides, it exhibited potent synergy when used with multiple antibiotic classes.^18^ Notably, MEDI3902 maintained the integrity of the lung, decreased bacterial burden, and shunned the dissemination of bacteria into the spleen and kidneys.^18^, ^140^ The safety, PK, and anti-drug antibody responses of MEDI3902 were assessed in a phase I dose-escalation study with healthy adults (ClinicalTrials.gov: NCT02255760). Following a single i.v. infusion, no severe treatment-emergent adverse events were observed other than infusion-associated reactions (e.g., redness and skin rashes).^140^ The small sample size was the major point of the current study, due to which, some less common safety events might not have been recognized.^140^ MEDI3902 is currently in a phase IIb clinical trial evaluating the efficacy and safety of MEDI3902 in 286 mechanically ventilated patients for the prevention of nosocomial pneumonia caused by *P. aeruginosa* (EVADE; ClinicalTrials.gov: NCT02696902). The preliminary results from the current trial showed that a single i.v. infusion of 1,500 mg MEDI3902 provided a mean serum concentration beyond the target level through day 22. Although anti-drug antibodies were detected in some subjects, they had no obvious effect on MEDI3902 PK. Consequently, the Data Monitoring Committee had no concerns with the safety data, and the results supported more MEDI3902 development (Guo et al., 2018, ECCMID, abstract). The scFv-based bsAbs in clinical trials are summarized in Table 2.

**Table 2:** The scFv-Based bsAbs in Clinical Trials and the Market

bsAb	Format	Targets	Biological Activity	Status (ClinicalTrials.gov)	Indication	Sponsor	Comments
MT110	BiTE	EpCAM/CD3	redirecting T cells to EpCAM-positive tumor cells	phase I	–	–	–
				NCT00635596	R/R EpCAM-positive solid tumors	Amgen	Despite the antitumor effects in two patients with ovarian cancer, DLTs, such as severe diarrhea and elevated liver enzymes, impeded dose escalation to therapeutic levels.
MEDI-565	BiTE	CEA/CD3	redirecting T cells to CEA-positive tumor cells	phase I	–	–	–
				NCT01284231	GI adenocarcinoma	MedImmune	DLTs, such as hypoxia (in two patients) and cytokine release syndrome (in one patient), no objective responses, and stable disease (28%) were observed in patients.
				NCT02291614	R/R GI adenocarcinoma	Amgen	A therapeutic window was not defined for MEDI-565, due to immunogenicity limiting adequate exposure for objective responses.
				NCT02760199	R/R GI adenocarcinoma	University Medical Center Groningen	–
BAY2010112	BiTE	PSMA/CD3	redirecting T cells to PSMA-expressing cells	phase I	–	–	–
				NCT01723475	prostate cancer	Bayer	–
AMG 330	BiTE	CD33/CD3	redirecting T cells to CD33-positive AML cells	phase I	–	–	–
				NCT02520427	R/R AML	Amgen	A complete response was seen in two patients who were on 240 μg/day.
AMG 420	BiTE	BCMA/CD3	redirecting T cells to BCMA-positive MM cells	phase I	–	–	–
				NCT02514239	R/R MM	Boehringer Ingelheim	It exhibited a favorable antitumor activity, and the objective response rate was 83% at the dose of 400 μg/day. Treatment-related serious adverse events were cytokine release syndrome and peripheral polyneuropathy observed at the dose of 800 μg/day, and no DLTs were reported up to 400 μg/day.
AMG 596	BiTE	EGFRvIII/CD3	redirecting T cells to EGFRvIII-expressing cells	phase I	–	–	
				NCT03296696	glioblastoma expressing mutant EGFRvIII	Amgen	–
AFM11	TandAb	CD19/CD3	redirecting T cells to CD19-positive tumor cells	phase I	–	–	Both trials were stopped due to serious adverse events, including one death and two life-threatening events in patients with ALL and NHL enrolled in the highest dose cohorts of each study, respectively.
				NCT02848911	RR adult B-precursor ALL	Affimed	
				NCT02106091	R/R CD19-positive B cell NHL	Affimed	
MGD011	DART-Fc protein	CD19/CD3	redirecting T cells to CD19-positive cells	phase I	–	–	–
				NCT02454270	RR B cell malignancies	Janssen Research & Development	Janssen terminated the enrollment of the trial due to clinical concerns for neurotoxicity observed in a number of patients receiving treatment.
PF-06671008	DART-Fc protein	P-cadherin/CD3	redirecting T cells to P-cadherin-positive tumor cells	phase I	–	–	–
				NCT02659631	P-cadherin expressing TNBC, CRC, or NSCLC	Pfizer	–
MGD006	DART	CD123/CD3	redirecting T cells to CD123-positive AML cells	phase I	–	–	–
				NCT02152956	RR AML or intermediate-2 or high-risk MDS	MacroGenics	The preliminary data revealed that MGD006 was well tolerable and exhibited anti-leukemic activity in patients at doses of ≥500 ng/kg/day.
				phase II	–	–	–
				NCT03739606	CD123-positive advanced ALL and other hematological malignancies	City of Hope Medical Center	–
MGD010	DART	CD32B/CD79B	inhibition of BCR-induced proliferation and Ig production in activated B cells	phase I	–	–	–
				NCT02376036	healthy subjects	MacroGenics	The results exhibited that MGD010 had an immunomodulatory activity with a satisfactory safety profile.
AFM13	TandAb	CD30/CD16A	redirecting NK cells to CD30-positive tumor cells	phase I	–	–	–
				NCT01221571	R/R classical HL	Affimed	Clinical results from this study confirmed the safety, tolerability, and therapeutic activity of AFM13 in R/R HL patients.
				phase II	–	–	–
				NCT02321592	RR HL	University of Cologne	AFM13 had efficacy as monotherapy in a subset of heavily pretreated subjects.
				phase Ib	–	–	
				NCT02665650	RR HL	Affimed	The clinical results of this study demonstrated that the combination of AFM13 and pembrolizumab was a well-tolerated treatment in RR HL patients.
				phase Ib/IIa			
				NCT03192202	RR cutaneous Lymphoma	Ahmed Sawas	AFM13 as monotherapy had a satisfactory safety profile and therapeutic activity in enrolled patients.
MM-111	scFv_2_-HSA	HER2/HER3	blocking HER2 and HER3 on HER2-overexpressing tumor cells	phase I	–	–	
				NCT00911898	advanced HER2-positive cancers	Merrimack Pharmaceuticals	–
				NCT01097460	advanced HER2-positive cancers	Merrimack Pharmaceuticals	–
				NCT01304784	advanced HER2-positive cancers	Merrimack Pharmaceuticals	The results proved the clinical activity of MM-111 and SOC HER2-directed regimens in patients with an overall clinical benefit rate of 52%.
				phase II	–	–	–
				NCT01774851	HER2-positive carcinomas of the distal esophagus, gastroesophageal junction, and stomach	Merrimack Pharmaceuticals	This study was terminated due to the low efficacy of MM-111 plus trastuzumab and paclitaxel in enrolled patients.
MM-141	anti-IGF-1R-IgG-scFv_2_	HER3/IGF-1R	blocking of IGF-1R and HER3, leading to IGF-1R-mediated growth inhibition	phase I	–	–	–
				NCT01733004	advanced solid tumors	Merrimack Pharmaceuticals	Patients could tolerate MM-141 (as a monotherapy or in combination with chemotherapy). The assessment of tumor specimens from treated patients exhibited the low cell surface levels of IGF-1R and HER3, demonstrating receptor internalization due to MM-141 activity.
				phase II	–	–	–
				NCT02399137	metastatic pancreatic cancer	Merrimack Pharmaceuticals	Results showed that the combination of MM-141 plus nab-paclitaxel and gemcitabine was not more effective than nab-paclitaxel and gemcitabine alone in enrolled patients.
MEDI3902	Fab_2_-scFv_2_-Fc	PcrV/Ps1	killing of *P. aeruginosa*	phase I	–	–	–
				NCT02255760	healthy adults	MedImmune	Following a single i.v. infusion, no severe treatment-emergent adverse events were observed other than infusion-related reactions, such as skin rashes with or without pruritus.
				phase IIb	–	–	–
				NCT02696902	nosocomial pneumonia caused by *P. aeruginosa*	MedImmune	The Data Monitoring Committee had no concerns with safety data, and the results supported more MEDI3902 development.
Blinatumomab	BiTE	CD19/CD3	redirecting T cells to CD19-positive B cells	approved	RR Philadelphia chromosome (Ph)-negative and Ph-positive precursor B cell ALL	Amgen	–

ALL, acute lymphoblastic leukemia; AML, acute myeloid leukemia; BCMA, B cell maturation antigen; BCR, B-cell antigen receptor; BiTE, bispecific T cell engager; bsAbs, bispecific antibodies; CEA, carcinoembryonic antigen; CRC, colorectal cancer; DART, dual-affinity retargeting molecule; DLTs, dose-limiting toxicities; EGFRvIII, epidermal growth factor receptor variant III; EpCAM, epithelial cell adhesion molecule; Fab, fragment antigen binding; GI, gastrointestinal; HER2, human epidermal growth factor receptor 2; HER3, human epidermal growth factor receptor 3; HL, Hodgkin lymphoma; HSA, human serum albumin; Ig, immunoglobulin; IGF-1R, insulin-like growth factor-1 receptor; i.v., intravenous; MDS, myelodysplastic syndromes; MM, multiple myeloma; NHL, non-Hodgkin’s lymphoma; NK cells, natural killer cells; NSCLC, non-small-cell lung cancer; *P. aeruginosa*, *Pseudomonas aeruginosa*; PcrV, type III secretion system protein PcrV; PK, pharmacokinetics; PSMA, prostate-specific membrane antigen; R/R, relapsed and/or refractory; RR, relapsed or refractory; scFv, single-chain fragment variable; SOC, standard of care; TandAb, tandem antibody; TNBC, triple-negative breast cancer.

### The scFv-Based bsAbs in the Market

Blinatumomab (Blincyto; developed by Amgen) is a first-in-class BiTE antibody recruiting cytotoxic T cells to kill CD19-positive ALL blasts.^142^ This BiTE antibody was approved by the US FDA for relapsed or refractory Philadelphia chromosome (Ph)-negative and Ph-positive precursor B cell ALL and by the EMA just for the treatment of relapsed or refractory Ph-negative precursor B cell ALL (Table 2).^143^

By evaluating data from six phase I and II trials in patients with relapsed or refractory ALL, minimal residual disease-positive ALL, and NHL, Zhu et al.^29^ demonstrated that blinatumomab has linear PK under cIV infusion over 4–8 weeks and fast clearance due to the lack of the Fc part and glycosylation. They also proposed that the loss of the Fc region, as well as the prevention of B cell differentiation into plasma cells (as the source of generation of anti-drug antibodies), might be the key causes leading to low immunogenicity of blinatumomab in patients. Their results showed that the type of cancer (ALL or NHL) or patients’ demographics had no clinically meaningful effects on blinatumomab PK. Furthermore, they highlighted that blinatumomab PD is involved in B cell depletion and dose-dependent cytokine elevation through T cell redistribution and activation.^29^ Aside from side effects, such as the cytokine release syndrome and neurological toxicities, not only do some patients not respond to blinatumomab, but some patients also experience disease progression during treatment second to the primary response.^144^ The escape of CD19-negative leukemia cells, overexpression of PD-L1 on leukemia cells, and increased numbers of regulatory T cells in combination with an elevated level of lactic dehydrogenase are the possible mechanisms causing the inefficiency of treatment with blinatumomab.^144^

More than 30 clinical trials (four phase III, two phase II/III, 21 phase II, four phase I/II, and seven phase I) are evaluating blinatumomab on different populations of patients with ALL, NHL, MM, Richter’s transformation, etc., as alone or in combination with chemotherapy or drugs, such as pembrolizumab, nivolumab, ipilimumab, ibrutinib, lenalidomide, etc. (clinicaltrials.gov).

### Conclusions

During the two recent decades, the bsAb technology, grabbing pharmaceutical companies’ attentions, has extensively progressed, particularly following the successful clinical use of blinatumomab in humans. Efficiently penetrating into tumor tissues and recruiting T cells and NK cells to kill tumor cells or blocking ligands involved in the pathogenesis of various disorders are some of the brilliant properties of bsAbs. To improve the PK profile of this generation of antibodies, different combinations of scFvs with other molecules, such as Fab, HSA, and the Fc part of IgG, were generated, resulting in the development of bsAbs with an extended half-life, potent activity, and more stability compared with other ones.

Therapeutic agents, such as rituximab, which not only is prescribed for patients with cancer but also is used in patients with autoimmune disorders, are inspirational in the development of antibodies used in a group of diseases.^145^ In this regard, finding targets, such as IL-1, IL-6, IL-17, IL-4, etc., which may be involved in both cancers and autoimmune diseases, can help design valuable agents used in a broad population of patients.^145^, ^146^, ^147^, ^148^ As the role of inflammation in the pathogenesis of cancer was established, designing more scFv-based bsAbs, such as SAR156597 (a DVD-IgG bsAb), which concurrently blocks IL-4 and IL-13 and is under investigation in two phase II trials (ClinicalTrials.gov: NCT02921971 and NCT02345070), can be a promising strategy to get rid of inflammation-related disorders.^148^, ^149^ The efficacy and safety of bsAbs were proven in patients with cancer and partly in patients with autoimmune diseases. However, due to the high rate of mortality in patients with infectious diseases, it is mandatory to focus on developing bsAbs targeting life-threatening bacteria. The development of bsAbs blocking vital proteins in the bacteria of the ESKAPE group or BiTE antibodies recruiting T cells against these bacteria can circumvent many problems in the health care system. The last, but not the least, the “cytokine storm” is one of the most dangerous side effects threatening patients receiving the BiTE antibody, such as blinatumomab. Therefore, the development of agents debilitating this life-threatening outcome can reduce concerns about BiTE therapy.

## Author Contributions

F.R.J. wrote the manuscript with support from all other authors.

## Conflicts of Interest

The authors declare no competing interests.
